# Managing Ramadan queries in COVID-19

**DOI:** 10.3399/bjgpopen20X101097

**Published:** 2020-05-18

**Authors:** Salman Waqar, Nazim Ghouri

**Affiliations:** 1 GP and Research Fellow, Nuffield Department of Primary Care Health Sciences, University of Oxford, Oxford, UK; 2 Consultant Endocrinologist and General Physician and Honorary Clinical Senior Lecturer, Institute of Cardiovascular & Medical Sciences, University of Glasgow, Glasgow, UK

**Keywords:** Consultation skills, Prescribing, Family medicine, Primary health care, General practice, Religious Beliefs

## Background

The holy month of Ramadan is a time of fasting from dawn to sunset for Muslims across the world. It is an important ritual in the lives of many, including those with chronic health conditions. Clinicians are frequently asked by patients for clinical advice and have little by way of guidance to inform their decision-making.

Here we outline some practical tips for clinicians on how to counsel and manage Muslim patients who are fasting in Ramadan, with some consideration for the context of COVID-19.

## What is Ramadan?

As one of the five pillars of the Islamic faith, the fast of Ramadan concurs with the ninth month of the Islamic calendar and is observed by the majority Muslims across the globe.^1^ In the UK, nearly 3 million British Muslims refrain from food and drink between dawn and sunset, which in the summer months can be a period of up to 18 hours.

Fasting is considered an obligatory ritual for all healthy adults, with exceptions given for certain groups, including those who are advised that they will come to harm from fasting owing to an acute illness or from complications related to an existing chronic condition.^2^

## Public health of Muslims in Western societies

It is important to note that Muslims in Western societies are predominately from black, Asian, and minority ethnic (BAME) backgrounds, and there is evidence showing chronic conditions are poorly managed in this patient population.^3^ BAME communities may also have lower medication concordance due to societal barriers.^4^ COVID-19 is having a disproportionate effect on BAME communities in Western nations; the reasons for this remain unclear but are thought to be multifactorial.^5^

## Risk stratifying patients

We have carried out a series of rapid evidence reviews and formed recommendations from experts who have experience of managing patients fasting in Ramadan.^6^ This has been done considering the uncertainty that COVID-19 poses, and a general need for such guidance. These recommendations are informative and do not form a directive.

We utilised a three-tiered risk assessment, based on the widely used criteria established by the Diabetes and Ramadan International Alliance, to form recommendations.^7^ Supplementary Table S1 can be used by healthcare professionals to assign a risk level and provide fasting advice accordingly. Patients in the two higher tiers, ‘very high risk’ and ‘high risk’, should receive medical advice that they ‘must not fast’ and ‘should not fast’ respectively. Those in the ‘low/moderate risk’ category are advised that the medical advice is on the discretion of the physician, along with the ability of the individual to tolerate the fast. Multiple comorbidities will likely be compounding and could upgrade the patient’s risk category. COVID-19 may pose additional risks, either directly or indirectly from the effect on healthcare services, and as a result some conditions may be reclassified as being higher risk.

Currently, there is no evidence or predictions to suggest that people who are healthy (that is, who do not have any diagnosed medical conditions) and who were previously able to observe the fast of Ramadan without any harm, are at any additional risk from fasting in the context of COVID-19, which is affirmed by the WHO interim guidance on Ramadan.^8^

Clinicians should be aware that there is a difference of opinion among Muslim jurists on whether inhalers and nebulisers break the fast.

## Managing acute illness

Muslims are religiously exempt from fasting if there is a reasonable fear that fasting will lead to harm, or if their recovery will be delayed by fasting. Each person would evaluate their circumstance on an individualised basis in accordance with the degree of symptoms they are experiencing, and any risks associated with fasting during that illness. Broadly speaking, this is judged on the prior experience of such an illness, common knowledge, and clinical advice.

In the context of a COVID-19 pandemic this presents challenges, as there is much we do not know about the virus, and many individuals will experience mild symptoms that do not need medical attention. In these patients, there is no evidence to suggest fasting would be deleterious and thus it may well be possible. Again, this needs to be judged individually based on pre-existing risk factors.

However, patients with fever and prolonged illness secondary to COVID-19 can become severely dehydrated and are at risk of sudden acute deterioration. As such, these patients should not fast (or cease fasting) and ensure adequate hydration.^9^ Further caution should be taken where other comorbidities are present.

Patients who have experienced an acute illness may resume fasting once they have made a recovery. They may seek clinical advice about when to restart fasting. This would be dependent on the risk of relapsing into illness, their ability to tolerate the fast, and if their recovery will be delayed by fasting.

## Managing chronic conditions

**Figure 1.**
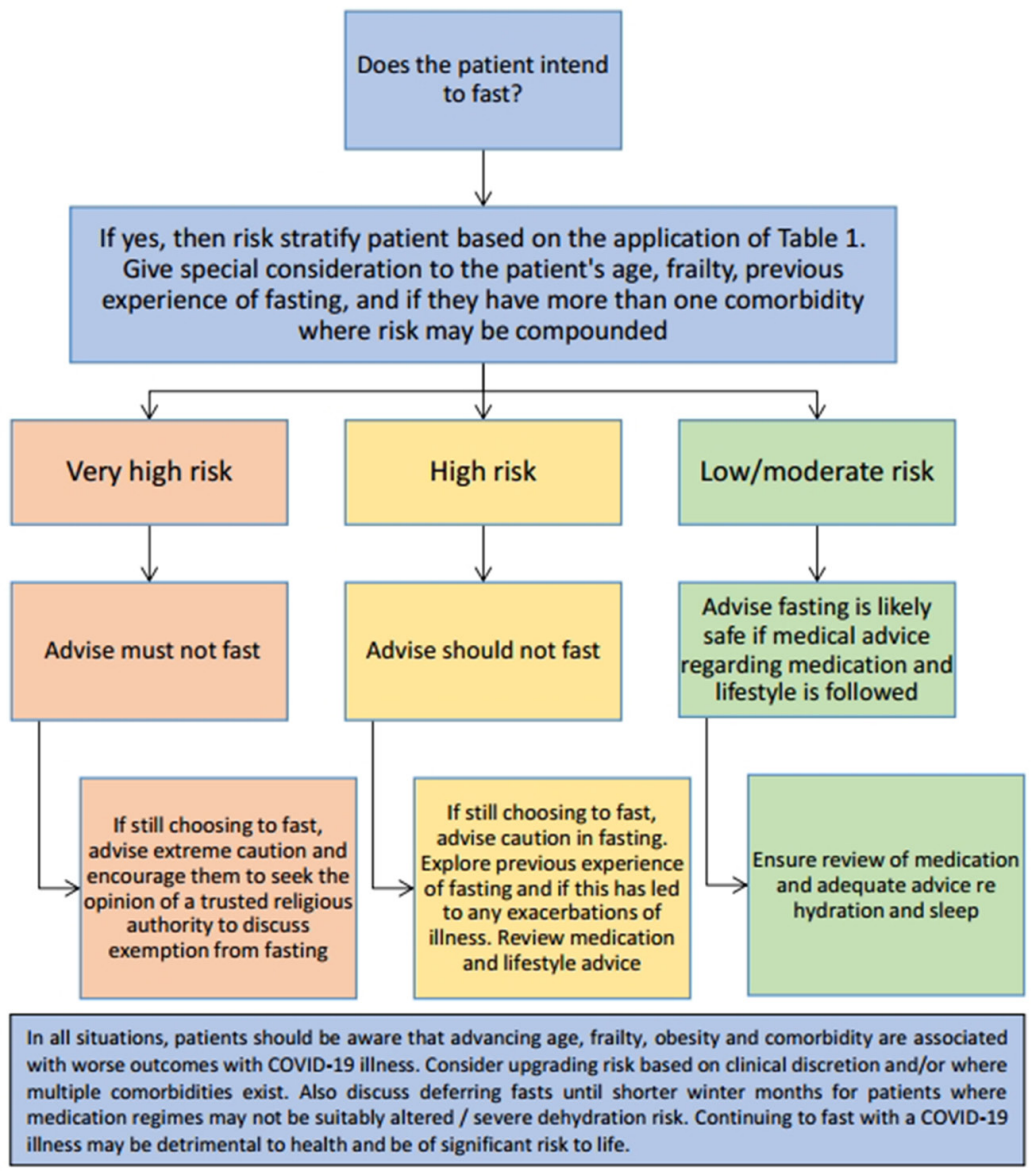
Decision making tool for those fasting with chronic conditions

Patients should be asked if they intend to fast and then risk stratified according to the severity of their condition, as per Supplementary Table S1, and advised accordingly. This is based on clinical discretion considering age, frailty, previous experience of fasting, multiple comorbidities, and any other factors the GP deems important. Figure 1 may be used by clinicians to help stratify patients.

Patients may be reluctant to heed clinical advice to the contrary, and if so, should be counselled with the following suggestions:

- Explore their experience of fasting previous years, especially if that had led to an exacerbation of illness, as well as their motivation for fasting against clinical concerns.
- Discuss the importance of medicines optimisation. Focus on the practicability of dosing regimes and the sequalae of events if chronic conditions progress out of control.
- Discuss fasting during the shorter winter months (where fasting duration is around 11 hours in the UK), or fasting a few days a week as alternative options.
- Advise seeking the opinion of a trusted religious authority, if preferred, for pastoral and religious guidance to reassure them that clinical concern is a valid religious contraindication to fasting. Here they can explore making up these fasts in the shorter winter months. If their condition is severe enough, they may have an exemption from fasting.

If, despite all effort, patients choose to fast against clinical advice they should still be supported. Advise vigilance in monitoring their health while fasting (for example, blood sugar, blood pressure, and weight); adequate sleep; and, where safe, adjust medication regimes. The importance of hydration in non-fasting hours should be emphasised, as well as having a low threshold for breaking the fast if experiencing any adverse effects.

For most Muslims, Ramadan remains a month of spiritual gains and has been associated with a positive effect on mental wellbeing.^10^ It would be premature to suggest that Ramadan would have a similar effect in the current COVID-19 pandemic, but some may still draw comfort from observing it.

## Conclusion

Fasting in Ramadan is an individualised choice and should be supported through a shared decision-making process. Behavioural change and activation during Ramadan means it is also an opportunity to discuss smoking cessation, weight management, and healthier eating.

It is important that clinicians recognise that some Muslim patients have a very strong motivation to fast in Ramadan, even if they have significant comorbidities such as cancer or advanced organ disease. Ignoring or being indifferent to this may lead to patients and their families losing trust in their clinicians, and possibly coming to harm from self-management and not seeking further counsel when needing advice. However, if they are sensitively and appropriately counselled, patients can be supported to have a safer Ramadan and gain the benefits the month brings.
